# Synthesis of a Starchy Photosensitive Material for Additive Manufacturing of Composites Using Digital Light Processing

**DOI:** 10.3390/molecules27175375

**Published:** 2022-08-23

**Authors:** Sofiane Guessasma, Sofiane Belhabib, Ferhat Benmahiddine, Ameur El Amine Hamami, Sylvie Durand

**Affiliations:** 1 INRAe, Research Unit BIA UR1268, Rue Geraudiere, F-44316 Nantes, France; 2 Université de Nantes, Oniris, CNRS, GEPEA, UMR 6144, F-44000 Nantes, France; 3 LaSIE, UMR CN 7356- La Rochelle Université, Avenue Michel Crépeau, CEDEX 01, F-17042 La Rochelle, France

**Keywords:** digital light processing, photosensitive starchy composite, 3D printing, microstructure, mechanical performance, thermal properties

## Abstract

In this study, digital light processing (DLP) was used to achieve 3D-printed composite materials containing photosensitive resin blended with starch and hemp fibers. The synthesis of 3D-printed composites was performed without heating, according to various material combinations ranging from pure photosensitive resin to a mixture of three phases, including resin, starch, and hemp fibers, with the weight content for each reinforcing phase reaching up to a third of the formulation. The morphology, composition, and structure of the 3D-printed composites were assessed using infrared spectroscopy, laser granulometry, X-ray diffraction, and optical and scanning electron microscopy. In addition, thermal behavior and mechanical performance were studied using calorimetry, differential scanning calorimetry, and tensile testing combined with high-speed optical imaging. The results showed that the post-curing step is a leading factor for improving the mechanical performance of the 3D-printed composites. In addition, hemp fiber or starch did not alter the tensile strength. However, the largest reinforcing effect in terms of stiffness improvement was obtained with starch. Additionally, starchy composites demonstrated the strongest dependence of heat capacity on operating temperature.

## 1. Introduction

Additive manufacturing (AM) is one of the major breakthrough technologies that have received major attention in recent decades [1,2]. It is a promising processing technology for designing technical parts with a high degree of complexity [3]. The simple definition that is commonly agreed for AM is that it is a process of joining materials layer-by-layer from a digitalized model [4]. The feature of interest in AM is the local control of the material deposition [5]. This local control enables a full customization of the part with a weak dependence on tooling [6]. It also provides the ability to manufacture a new generation of materials, such as adaptive materials [7]. The short fabrication cycle that characterizes AM is an attractive technology for several sectors, such as the bioengineering, aeronautics, civil engineering, modelling, automotive, engineering, the food industry, and art sectors [8,9,10,11,12]. The emergence of AM is undoubtedly related to the large number of processes that fall within the definition of AM, enabling a large spectrum of materials to be printed [13]. For instance, fused filament technology or fused deposition modeling (FDM) is a popular and affordable way to print polymeric structures [14]. Laser-based technologies, such as selective laser melting (SLM), are AM processes that are used for dealing with metallic powders [15]. Stereolithography is another process that works with a laser source but targets photosensitive resins [16]. This technology features high-resolution processing, compared with fused filaments, and results in highly isotropic structures [17]. The basic principle behind stereolithography involves the use of a laser beam to scan the surface of a photosensitive resin in the liquid state [16]. Depending on the 2D pattern issued from the slicing step, the polymerization of the structure occurs at the spot targeted by the laser beam [18]. The high reachable resolution for stereolithography comes at the cost of a lengthy process because of the large toolpath scanning [19]. 

Digital light processing (DLP) is another AM route that shares the same rationale as stereolithography but solves the problem of long term scanning by using a digital screen that projects an image of the layer under construction [20]. Therefore, the DLP process has the ability to polymerize an entire layer instead of a single spot [21]. Products that are based on DLP are mainly used for prototyping, molding, and consumer applications [20,22].

In this work, we report for the first time a successful attempt to process starch and hemp fiber fillers in additive manufacturing using the photopolymerization route. Starch is considered as a solid filler in a sensitive resin, and the evaluation of the composite performance and the cost point are discussed. In fact, photosensitive resins are known to be expensive feedstock materials, and only a limited choice of materials can be processed. Blending can be a solution in designing new structures with specific performance requirements, such as water sorption/desorption capability, while maintaining a reasonable printing cost. In addition, the use of biomass has proven to be a strong trend in the plastics industry because of the environmental footprint, high specific mechanical properties, biocompatibility, and transfer properties. For instance, Azmin et al. [23] developed bioplastic films from agricultural waste with low water adsorption and low water vapor permeability. Fatima et al. [24] developed nontoxic a biocomposite material from bacterial cellulose for biomedical applications and showed the potential of these materials in terms of transfer properties, while addressing the environmental and economic costs. In this work, we show that starch filler can be a good choice because it does not lower the performance of the resin and provides good transfer properties. 

The other motivation behind this study was to bypass the difficulty of processing starch by using other AM routes. Indeed, starch is not suitable for FDM, as the heating of starch beyond the glass transition results in a large drop in mechanical stability, making it impossible to support any layering during the printing process.

## 2. Materials and Methods

### 2.1. Preparation Steps

**Figure 1:**
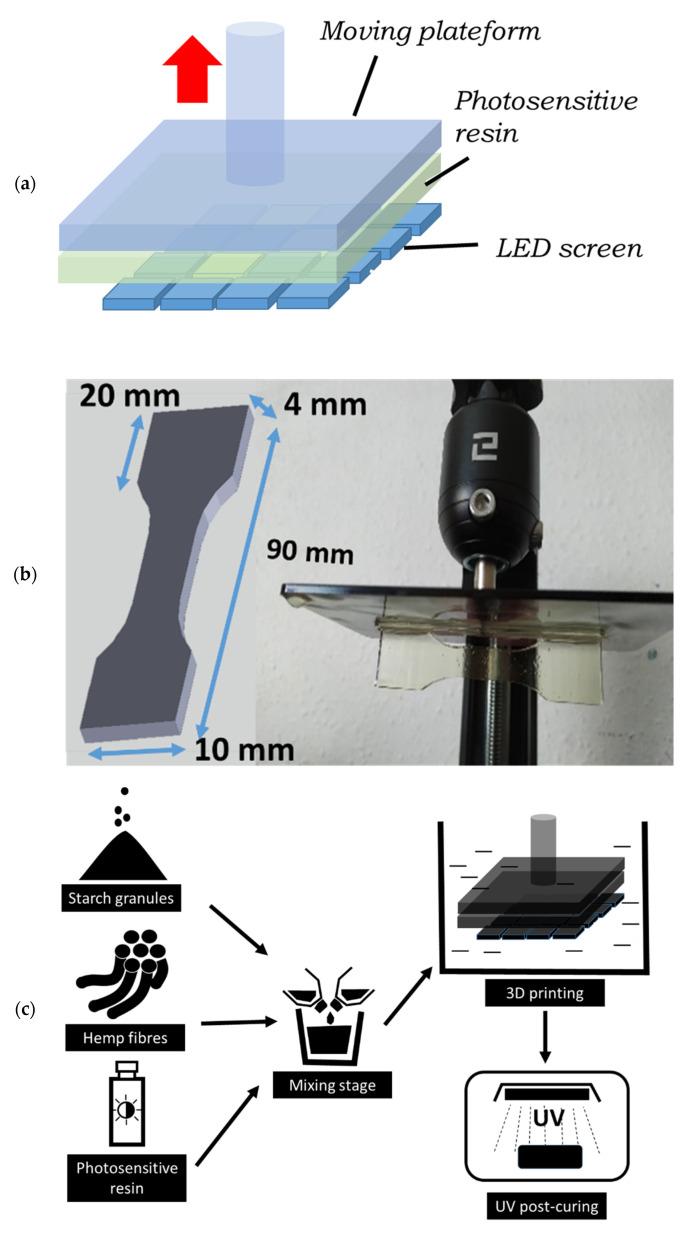
Digital light processing illustrated: (**a**) sketch describing the principle of DLP; (**b**) sample, dimensions, orientation, and printed dogbone sample using longer photosensitive resin; (**c**) illustrative sketch of the process preparation up to the post-treatment.

The DLP manufacturing process was performed using a commercial 3D printer that allowed for a build space of 120 × 68 × 155 mm^3^ (Figure 1a). The resolution of the equipment was directly correlated with the pixel size of the LED screen. In the present study, a pixel size of 47 µm was achieved under a 2 K (2560 × 1440 pixels) resolution. The accuracy of the *z*-axis positioning was 1.25 µm. Curing of the photosensitive resin was carried out using the UV light of the LED screen at a wavelength of 405 nm. The exposure time for the first five layers was kept at 20 s and lowered to 7 s for the remaining layers. The geometry of the printed structure was a typical tensile specimen with dimensions of 90 × 10 × 4 mm^3^, with a gauge length of 20 mm (Figure 1b). The printing of these structures was performed using either a 15 min UV or non-UV post-curing stage. The main steps of the preparation up to the posttreatment are shown in Figure 1c. The printing direction was aligned with the thickness of the specimen.

### 2.2. Experimental Materials

The feedstock materials used in this study comprised a standard transparent photosensitive resin (wavelength of 405 nm) purchased from the Longer company (Shenzhen, China) under the tradename of Longer (LNG). The physical properties of the resin are provided in Table 1. Native maize starch was used as a filler (STR). This was purchased from Unilever (London, UK), with a typical granulometry of 10 µm to 30 µm. According to Stasiak et al. [25], the preconsolidation shear stress of maize starch powder is of the order of 2.7 kPa, while the maximum shear stress reaches 3.25 kPa. According to the same study, under compression loading, the starch powder exhibits nonlinear behavior with typical maximum stress values of the order of 10 kPa. The volume fraction of starch was kept constant in all formulations. In addition to starch, natural fibers (FIBs) were used as the second type, and their volume content was kept constant. Details for the processing conditions and volume content for all phases (LNG, STR, and FIB) are provided in Table 2, leading to the formulation of four different composites. For instance, the composite LSC0 was formulated using the STR–LNG weight proportion of 1:2, which meant that for 100 g of composite, 33 g of starch was present. After processing, this composite was cured for 15 min. The printing duration for a single specimen was 20 min which, when compared to FDM under the same resolution, is eight times lower. Up to 12 samples per condition were printed for further analysis.

### 2.3. Instruments

Liquid resin and solid extract of the 3D-printed sample (LNG+STR) were analyzed by mid-infrared spectroscopy with a Thermo Nicolet IS50 spectrometer (Thermo Scientific, Courtaboeuf, France). The spectra were collected in reflexion mode between 4000 cm^−1^ and 400 cm^−1^ at 8 cm^−1^ resolution using a Smart iTX–ATR diamond accessory. The infrared spectra were obtained from 200 coadded scans using OMNIC 9.2.41 software (Thermo Scientific, Courtaboeuf, France). All spectra in the 4000 cm^−1^ to 700 cm^−1^ region were baseline-corrected and unit vector normalized, and the mean spectra were calculated for three repetitions using OPUS 7.5 software (Bruker Optics, Champs-sur-Marnes, France). A comparison of the spectra with those from the OPUS library enabled us to identify the main components present in the resin.

**Figure 2:**
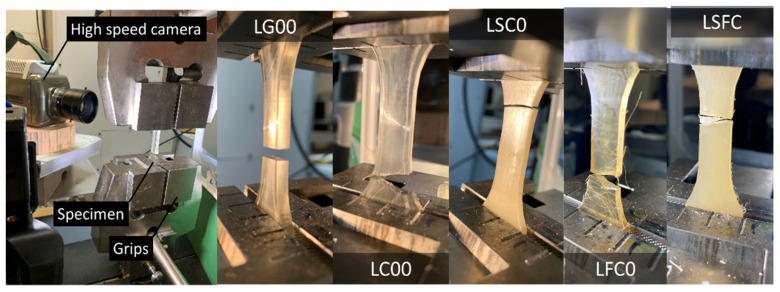
Tensile testing experiments showing the setup with high-speed camera monitoring and ruptured specimens for selected composites.

Mechanical testing was performed using a Zwick Roell universal machine equipped with a 10 kN load cell (Figure 2). A fixed displacement rate of 5 mm/min was applied up to the rupture point. Four replicates per condition were planned under these conditions. Tensile testing was monitored using a high-speed camera (Phantom V7.3 from Photonline, Marly Le Roi, 78-France). Sample deformation and rupture behavior was captured at different frame rates, pixel sizes, and resolutions typically from 50 fps–frames per second–135 µm, 800 × 600 pixels to 55,000 fps, 139 µm, 96 × 304 pixels.

The microstructure of the resin/starch composites and rupture patterns were observed using a scanning electron microscope (SEM) in environmental mode (FEI Quanta 200). With this mode, high-resolution observations were made without the need for prior treatment and preparation of the sample surface. This was possible due to the presence of a low pressure inert gas and/or water vapor in the sample chamber, avoiding vacuum stresses. To do so, an accelerated voltage of 20 kV with different levels of magnifications was selected. Images were obtained with a resolution varying from 1280 × 1024 pixels, corresponding to a physical pixel size from 0.11 µm to 3.13 µm. Samples were prepared by gold/palladium coating prior to observation.

The water vapor sorption isotherms for the studied materials were obtained by a volumetric method using the “Belsorp Aqua 3^®^ device”. The measurement principle for this device involved determining the quantity of adsorbed and desorbed water vapor by means of the acquisition of water vapor pressure and application of the ideal gas law. The measurements were performed at the equilibrium state for the samples and at a constant temperature of 23 °C. Prior to the test, samples with a height and diameter of 40 mm and 5 mm, respectively, were dried in a vacuum oven at 40 °C until their mass became stable.

The crystallinity analysis of the studied composites was carried out using the X-ray diffraction technique with INEL EQUINOX 600 X-ray equipment (France). The tested samples had the form of solid platelets. A copper (Cu) radiation source was used with a wavelength of 1.54060, voltage of 40 kV, and current intensity of 40 mA. The diffraction patterns were obtained after a 2 h exposure time and normalized with respect to the total scattering value. The 2θ range was selected between 5° and 50°. The thermal behavior of both starch powder and photosensitive composites was determined using differential scanning calorimetry (DSC). The thermoanalytical analysis was conducted using DSC3+ equipment from METTLER Toledo. Temperature scanning was carried out using the following temperature profile: the isotherm stage at 25 °C was maintained for 5 min, followed by a heating stage from 25 °C to 400 °C, with a heating rate of 10 °C/min under a controlled argon ambiance with a flow rate of 25 mL/min.

The heat capacity of the samples was measured using a Calorimeter Calvet^®^ for a temperature range between −10 °C and 45 °C and a speed of 0.1 °C/min. The major advantage of this device lies in the fact that it uses 3D fluxmetric sensors that completely surround the sample, which allows for a more accurate result. The morphology of the starch powder was determined using a numerical optical microscope (Keyence) with a typical resolution of 2880 × 2160 pixels. Observations were performed under different magnification levels (from 20× to 400×) with a pixel size varying from 0.26 µm to 5.2 µm. The size distribution of the starch granules was assessed using laser granulometry equipment (CILAS 1090), for which the measurement size range was 0.04 µm to 500 µm. The measurement of the size distribution was conducted under dry mode. Four replicates from different batches were used, and excellent reproducibility was achieved. 

## 3. Results and Discussion

### 3.1. Composition of the Photosensitive Resin

**Figure 3:**
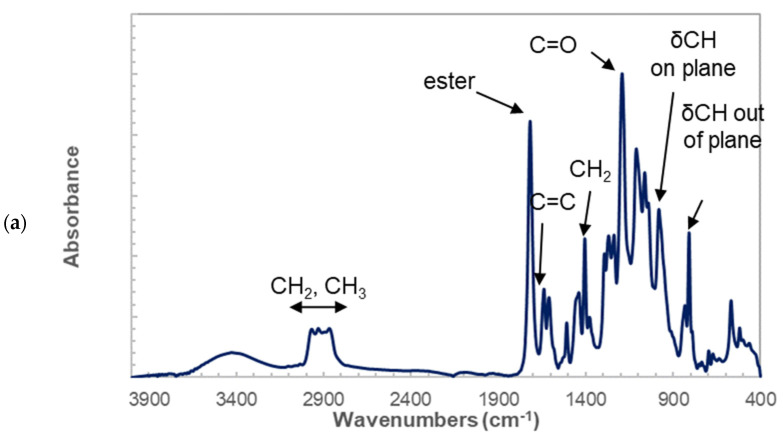

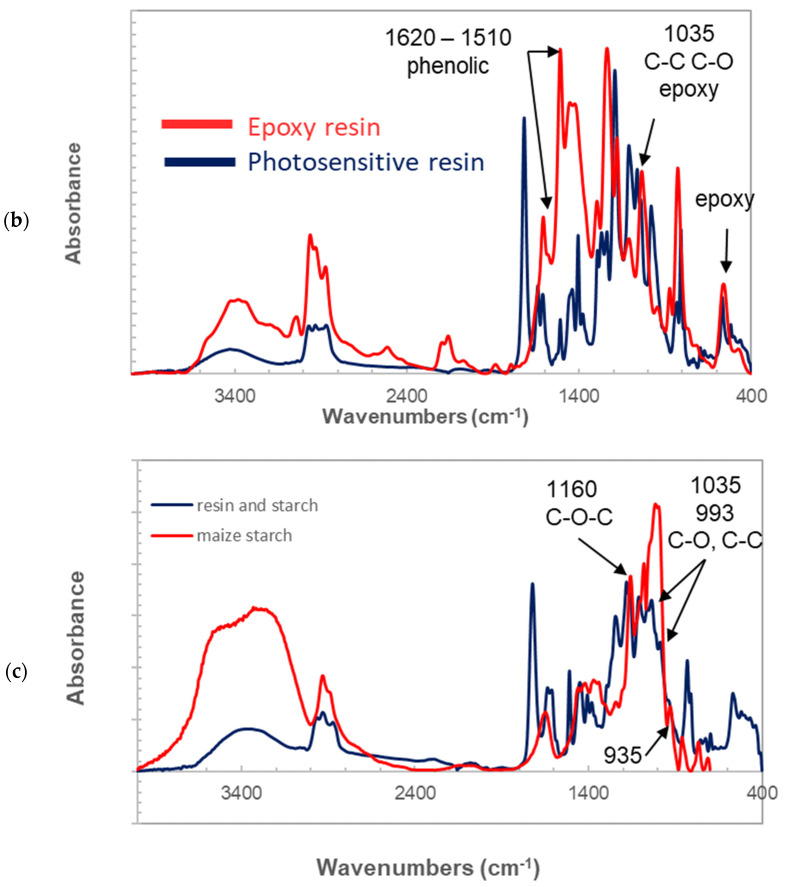
Analysis of photosensitive resin composition (**a**) Infrared spectrum of as-received photosensitive resin with band width assignment, (**b**) comparison between as-received and epoxy resin spectra, (**c**) testing experiments showing the setup with high-speed camera monitoring and ruptured specimens for selected composites.

Figure 3 shows the infrared spectra of both the photosensitive resin (LNG) in the liquid state and after polymerization with starch granules used as filler (LSC0). Figure 3a shows the band assignment for the as-received photosensitive resin. The analysis of the bending and stretching frequencies revealed common components in photosensitive resins [26], which are generally composed of a polymeric binder such as polybutadiene, a polymerizable unsaturated monomer such as ethylhexyl acrylate, a polymerization initiator such as benzoin alkyl ether or benzoin and a thermal polymerization inhibitor such as hydroquinone, and eventually pigments.

The main components present in the LNG resin are acrylic compounds, such as butyl-, ethyl- and methyl-acrylate in greater proportion.

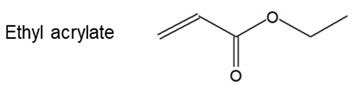
(1)

The infrared-assigned features characteristic of acrylic compounds are the ester C=O band at 1720 cm^−1^, the ethylenic stretching C=C vibration at 1640 cm^−1^, the CH_2_ deformation at 1460 cm^−1^, and the stretching C-O band at 1990 cm^−1^. The associated peaks at 982 and 810 cm^−1^ are assigned according to the in- and out-of-plane C-H bending.

On the other hand, comparing longer resin spectra with an epoxy resin spectrum (Figure 3b) for the following chemical formula.

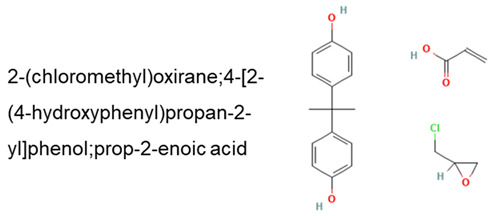
(2)

Revealed several common peaks: the aromatic C=C stretching vibrations bands at approximately 1600 cm^−1^ and 1515 cm^−1^, the C-O and C-C stretching bands of the epoxy group at 1035 cm^−1^, and the epoxy deformation bands at 570 cm^−1^.

Observation of the infrared response of LSC0 containing starch (Figure 3c) revealed polysaccharides bands corresponding to C-O-C osidic binding at 1160 cm^−1^, C-O and C-C stretching bands at 1035 and 993 cm^−1^ and C-CH, and C-OH deformation bands at 935 cm^−1^. The presence of starch did not alter the assignment identified for the photosensitive resin in Figure 3a. 

### 3.2. Photopolymerization Results

**Figure 4:**
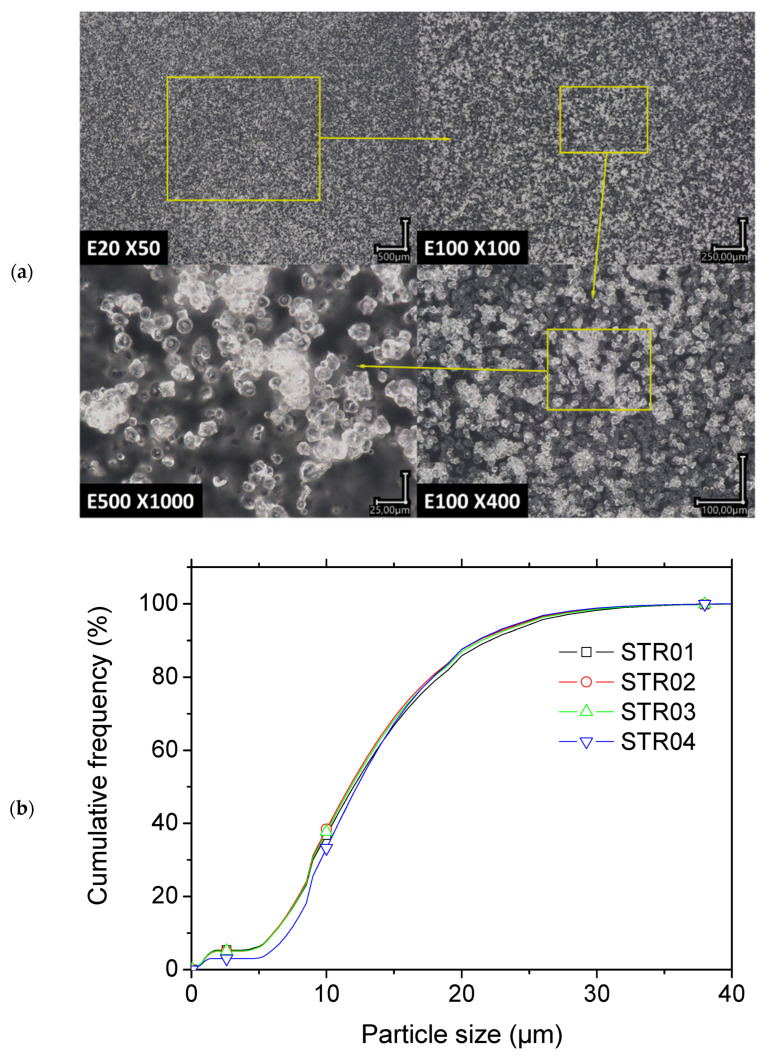

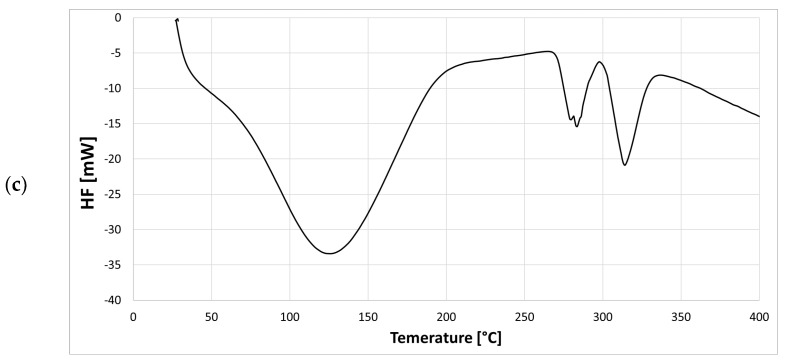
(**a**) Starch granule morphology from optical microscopy at different magnifications, and (**b**) particle size distribution prior to blending (**c**) calorimetric behavior of native maize starch.

Figure 4a exhibits the morphology for the starch granules used as a filler in the photosensitive resin. The optical micrographs suggested a globular form for starch, with a shape factor near 1. Clusters of starch granules formed as a result of excess water content. The results of further investigation of the dried starch granules that was aimed at determining the particle size are provided in Figure 4b. This figure shows the native starch particle distribution for four replicates. A typical bimodal size distribution was observed, with two average values of 1.2 µm and 14.0 µm, where the population weight of small particles represented approximately 4.68% of the entire particle population. Differences existed between the range provided by the supplier and the range determined from granulometry analysis. The typical size range provided by the supplier, between 10 µm and 30 µm, was captured by the analysis. However, larger particles representing 0.92% of the entire size population and smaller particles below 10 µm with a proportion of 29% of the entire distribution were reported by the supplier. Knowing that the printing resolution was dependent on a pixel size of 50 µm, the particle size was considered adequate so as not to alter the UV light polymerization, as long as the amount of starch was optimized.

In fact, around a wavelength of 410 nm, the absorbance of starch was limited, as shown by Wuttisela et al. [27]. The authors studied the absorbance of starch within the wavelength range of 400 nm to 800 nm and concluded that the maximum absorbance peak occurred at approximately 610 nm. On the other hand, Ryu et al. studied the absorbance of hemp within the wavelength range of 190 nm to 400 nm and showed the presence of absorbance peaks below 350 nm. Musetti et al. extended the study to a larger UV absorbance range between 200 nm and 600 nm and confirmed the absence of an absorbance beyond 350 nm. On the other hand, Ryu et al. [28] studied the absorbance of hemp within the wavelength range of 190 nm to 400 nm and showed the presence of absorbance peaks below 350 nm. Musetti et al. [29] extended the study to a larger UV absorbance range between 200 nm and 600 nm and confirmed the absence of absorbance beyond 350 nm. Thus, starch is the limiting factor for increasing the filler load, as it absorbs UV light at the same wavelength used for resin photopolymerization.

Preliminary tests conducted to increase the amount of starch from 10% to 40% in the formulation showed that the maximum amount of starch is approximately 30%. Beyond this limit, two main problems occur: the first issue is related to the drastic increase in the blend viscosity, which does not allow the printing platform to perform the prescribed height increments of 50 µm. This issue is further amplified by the starch sedimentation that creates a gradient for the starch content. The second problem is related to the UV absorbance of starch, which is estimated at the operating wavelength (355 nm to 410 nm) to be approximately 19% of the absorbance peak, according to Wuttisela et al. [27]. A higher load of starch alters the full polymerization of the composite.

This alteration is materialized by the loss at curing depth. Indeed, the polymerization depth depends on the laser intensity and exposure time. For a laser intensity of 5 × 10^4^ W/m², the polymerization depth of the pure resin can be as large as 70 µm, according to the model used by Sun et al. [18]. This depth increases to approximately 90 µm for a laser power of 25 × 10^4^ W/m². In the present study, with a spot size of 50 µm and an optical laser power of 10 W to 11 W, the estimated curing depth was 55 µm after 0.5 s of exposure. By combining an exposure time ranging from 7 s up to 22 s and a layer height of 50 µm, only minor alteration of the curing occurred, due to the presence of second phase material in the resin formulation as long as the starch load was below 40%. In fact, the main two phenomena altering the curing were incident beam absorbance by starch and photon scattering by either starch or hemp. The absorbance represented nearly one-fifth of the incident beam, and the lateral laser beam scattering resulted in the loss of 16% of the maximum depth for a load content of 30%, according to the model of Sun et al. [18] for an exposure time of 0.5 s and laser intensity of 5 × 10^4^ W/m². 

### 3.3. Properties of the Synthetized Photosensitive Starch

Figure 4c exhibits the thermogram for the native maize starch, where the total heat flux is determined within the range of 25 °C up to 400 °C. The DSC profile shows the main region of loss of absorbed and bound water with a peak observed close to 124 °C. A second domain above 250 °C corresponds to the decomposition of maize starch with a peak value close to 283 °C. For instance, Rachid et al. [30] reported peak values for the two domains close to 98 °C and 270 °C, respectively. The first domain masks the glass transition domain manifested by the inflection within the range of 60 °C to 70 °C for native maize starch; the enthalpy change corresponding to the glass transition was reported by Wang et al. [31] to be close to 77 °C. Thermogravimetric analysis conducted below the thermal degradation of starch showed the zone of water release covering the low temperature range between 25 °C and 200 °C and part of the medium range where the thermal stability for starch was observed (between 200 °C and 250 °C). The thermal transition profile achieved here was similar to the result obtained by Saraiva Rodrigues et al. [32], who studied the thermal transitions of babassu mesocarp starch using thermogravimetric analysis (TGA/DTG) and divided the thermal behavior into four main stages. The first stage corresponded to the release of water (between 20 °C and 125 °C); the second stage reflected the thermal stability of starch (between 15 °C and 240 °C); the third stage was between 240 °C and 330 °C and concerned the degradation of organic components; the last stage, in the high temperature range (330 °C to 500 °C), characterized the completion of starch degradation. The temperature for starch degradation depends on the type of starch. For instance, Moran et al. [33] showed a degradation peak for native potato starch at 309 °C. It can be concluded from the thermal analysis that starch remains thermally stable during the printing process, especially given that UV polymerization does not generate excessive heat.

**Figure 5:**
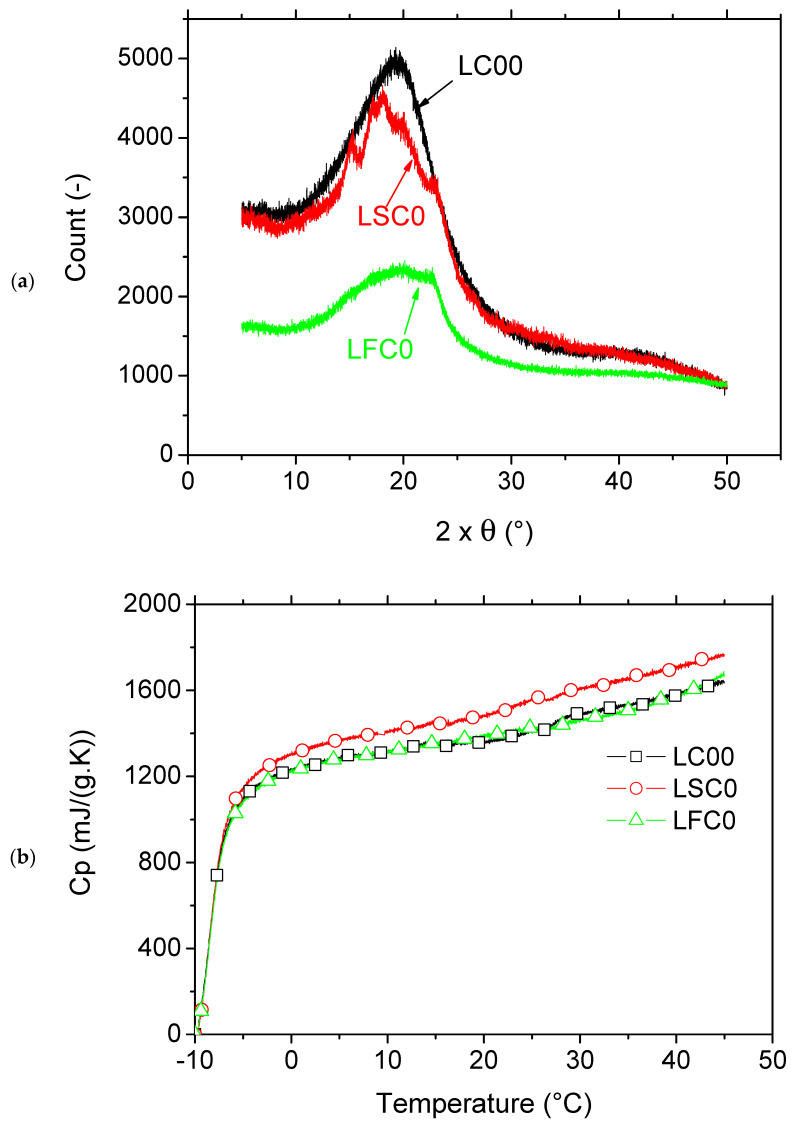
Comparison between genuine photosensitive and modified resins by starchy particles. (**a**) X-ray diffraction patterns, (**b**) specific heat capacity.

Figure 5a shows the X-ray diffraction pattern for genuine photosensitive resin (LC00), the same resin modified by adding starch material (LSC0) and natural fibers (LFC0), all of which were polymerized using UV and post-cured. The broad Gaussian shape observed for LC00 is typical of an amorphous structure consisting of a polymeric network that is formed upon the cross-linking process by UV activation. This network is mainly composed of a mixture of styrene monomers and oligomic acrylate. The presence of starch particles in the mixture modifies the Gaussian form by adding characteristic peaks at different 2θ angles, as shown by Manek et al. [34]. These peaks are related to the semicrystalline structure of starch that has not yet been thermally transformed. These typical peaks lie at 2θ values of 15°, 17°, and 23° with a shoulder at 18°. The starch structure corresponds to a typical A-type pattern [35]. The additional crystallinity added to the mixture is believed to further improve the mechanical performance of the LSC0 composite. This particular observation is discussed later, in light of the tensile results obtained for these materials. The last formulation considering the natural fiber addition (LFC0) did not allow for the capture of any crystallinity, although native crystalline cellulose was still present, as barely evidenced by a small (002) peak at 2θ of approximately 23° [36].

The thermal properties of all modified and genuine resins were compared according to the evolution of the specific heat capacity as a function of temperature (Figure 5b). The results showed that both resins exhibited the same behavior at temperatures below 7 °C. At higher temperatures, LSC0 exhibited a higher Cp, which can be explained by the heat capacity dependence of starch. Indeed, Louaer et al. [37] determined the Cp profiles for maize starch within a large range of temperatures covering the glass transition and the gelatinization domains (0 °C to 150 °C) under hydrated and dehydrated conditions. The authors reported an increase in Cp with increasing temperature up to an inflection point close to 107 °C, which depended on the water content. Their result corroborated the fact illustrated in Figure 5b, that Cp for LSC0 continuously increased within the range of 0 °C to 50 °C.

**Figure 6:**
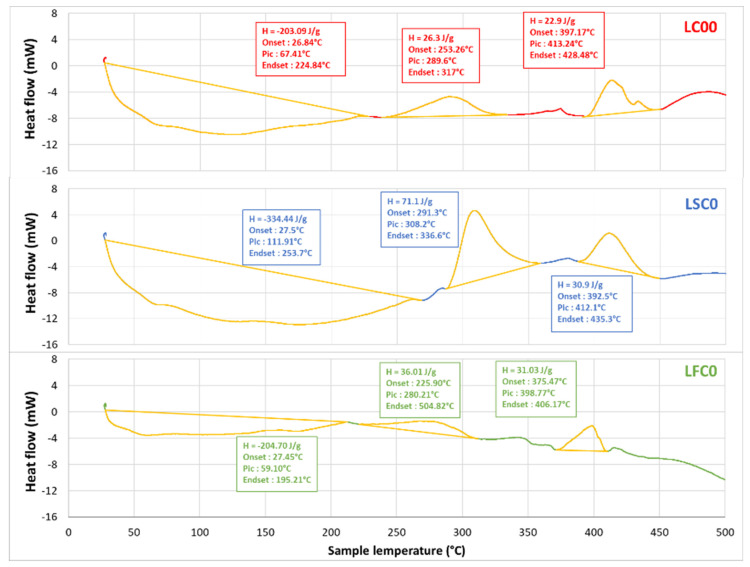
DSC results of photosensitive composites.

Figure 6 shows the DSC results for the LC00, LSC0, and LFC0 composites. The main thermal transitions for the photosensitive resin showed both endothermal and exothermal effects, where three main peaks were identified. The first peak corresponded to the glass transition for the cured resin, which overlapped with the enthalpy relaxation and post-curing reactions. The peak corresponding to the glass transition was found close to 112 °C. This stage was followed by softening and partial melting of the resin, thermal instabilities related to volatiles, and decomposition stages at temperatures above 250 °C. Composites formulated with native starch granules introduced other thermal transitions related to the organic behavior of starch, identified in Figure 4c, related to water release and organic decomposition. These events overlapped with the thermal transitions for LC00 and consequently led to a shift in the first and second peaks toward large temperatures by at least 10 °C, as shown in Table 3. The third peak remained stable as the decomposition of starch was completed at temperatures above 330 °C. The blending of the photosensitive resin with natural fibers (LFC0) also revealed characteristic peaks, due to hemp. In addition to the modifications observed at low temperatures, which were also genuine to the presence of hemp, such as water uptake, the thermal behavior at high temperatures also superposed the decomposition of cellulosic material within the temperature range of 280 °C to 500 °C, as shown by Stevulova et al. [38] via DSC analysis of hemp and hemp composites. In the present case, this consequently also shifted the third peak toward lower temperatures, due to hemp degradation.

### 3.4. Mechanical Testing Results

**Figure 7:**
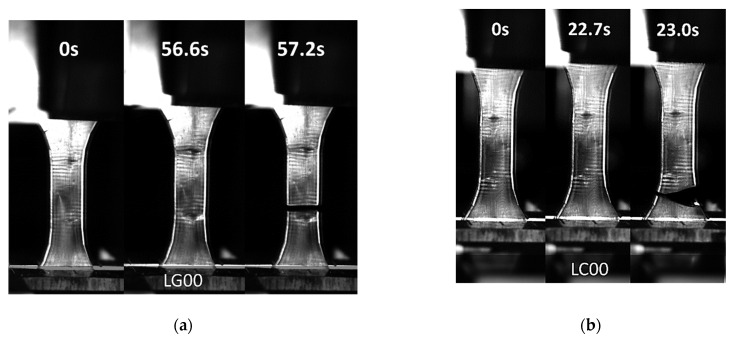

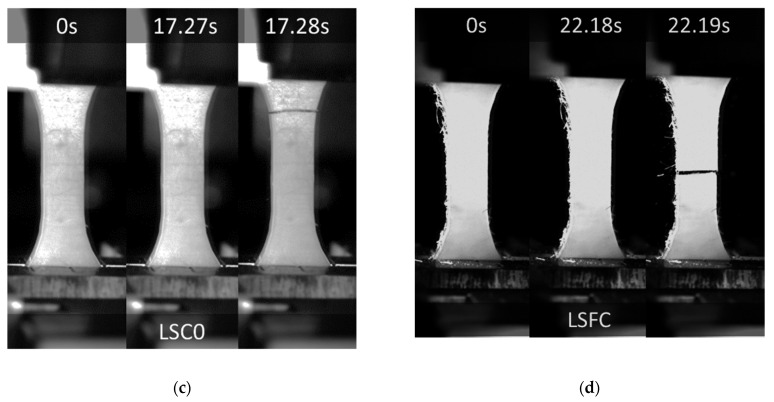
Deformation sequence of studied composites captured at different times: (**a**) LG00 (**b**) LC00, (**c**) LSC0, (**d**) LSFC.

Figure 6 shows the deformation sequences for the studied composites acquired by high-speed camera recording. Figure 7a,b illustrates the effect of the curing duration on the rupture sequence of the photosensitive resin without filler (LC00 and LG00 formulations). Figure 7a suggests a larger elongation at break for the non-cured sample. A significant brittleness was observed for the cured resin, with a tendency to fragment at the rupture point (Figure 7b). Examination of the engineering constants provided in Table 4 demonstrates that curing has the largest positive effect on both tensile strength and stiffness, while it has a negative effect on the elongation at break. Indeed, the post-curing step was responsible for 144% of the increase in Young’s modulus and 14% of the increase in the tensile strength. The elongation at break was lowered by 67%.

**Figure 8:**
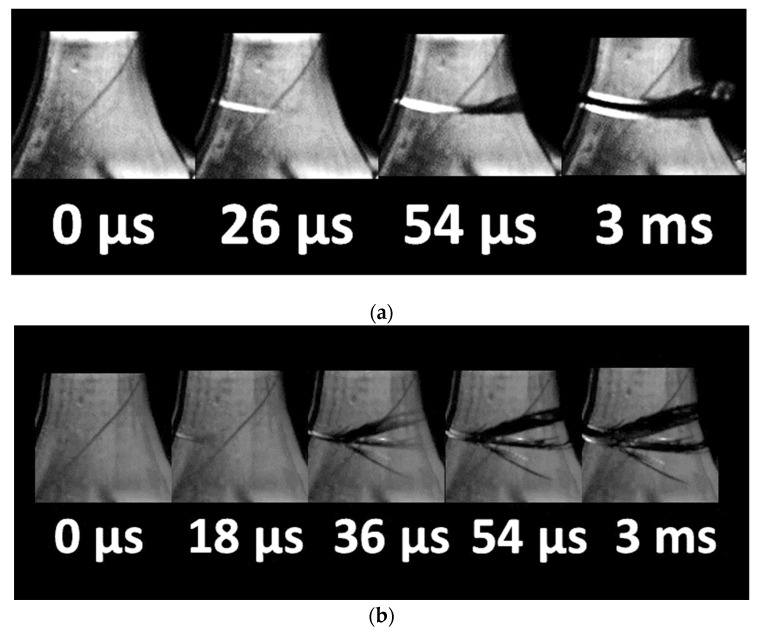
Crack propagation in printed composites (**a**) LG non-cured state, (**b**) LC cured state.

The addition of starch or hemp fiber led to the same overall brittle behavior for the cured resin, as evidenced by the optical micrographs recorded during the tensile testing (Figure 7c,d). It must be mentioned that, depending on the natural fiber amount, significant delays in cracking can be expected. Additionally, the addition of second phase particles that are weakly bonded to the matrix can lead to cracking jaggedness and rupture profiles that deviate from the opening mode. However, this behavior is not captured in Figure 7c,d, and instable cracking remains the prevailing behavior. The cracking behavior was further examined through optical micrographs acquired by high-speed recording (Figure 8). Quasi-brittle cracking behavior was observed, even for the uncured samples. A typical propagation speed of 265 m/s was achieved, with a marked tendency for branching observed for both uncured and cured photosensitive resin samples (Figure 8). This branching led to fragmentation at the failure point. For cured samples, extensive branching is depicted, with larger propagation speeds of 497 m/s.

**Figure 9:**
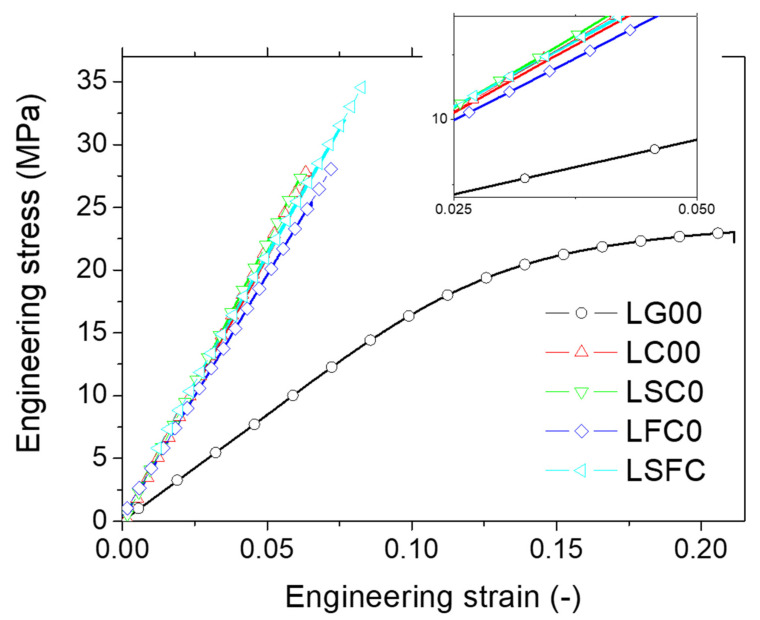
Tensile response of the printed samples as a function of DLP printing conditions.

Examination of the tensile response (Figure 9) showed that green (uncured) samples have a significant plasticity stage. This plasticity disappeared when the post-curing step was added. The curing resulted in nearly elastic composites with improved stiffness and tensile strength. However, slight differences were observed between the composites. For instance, both the LC00 and LSC0 formulations exhibited the top-ranked behavior. The average engineering constants summarized in Table 4 from all replicates promoted the LSC0 formulation as the one that resulted in 8% improvement of stiffness, compared with the cured genuine resin (LC00). On the other hand, the addition of hemp fiber lowered both the stiffness and strength by 4%. Finally, the addition of starch to the resin-fiber composite did not lead to the expected positive effect on stiffness. However, the three-phase material LSFC exhibited a higher tensile strength (i.e., 4% improvement), despite the relatively higher discrepancy in the results. For all cured composites, there was no strong tendency with regard to the elongation at break, and the only significant reduction observed of 70% was related to the post-curing stage, irrespective of the type of filler used for blending. This meant that both hemp and starch fillers did not alter the curing process, despite the differences in shapes and sizes. It can be stated that the achieved responses shown in Figure 9 and the data reported in Table 4 promoted curing as the factor responsible for the observed significant increase in stiffness and strength. While the quality of the hemp–resin/starch–resin bonds played an important role in load transfer, the interface properties may be regarded as a limiting factor if the bond is weak, and the improvement of the tensile performance was more related to the phase intrinsic properties.

### 3.5. Microstructural Arrangement

**Figure 10:**
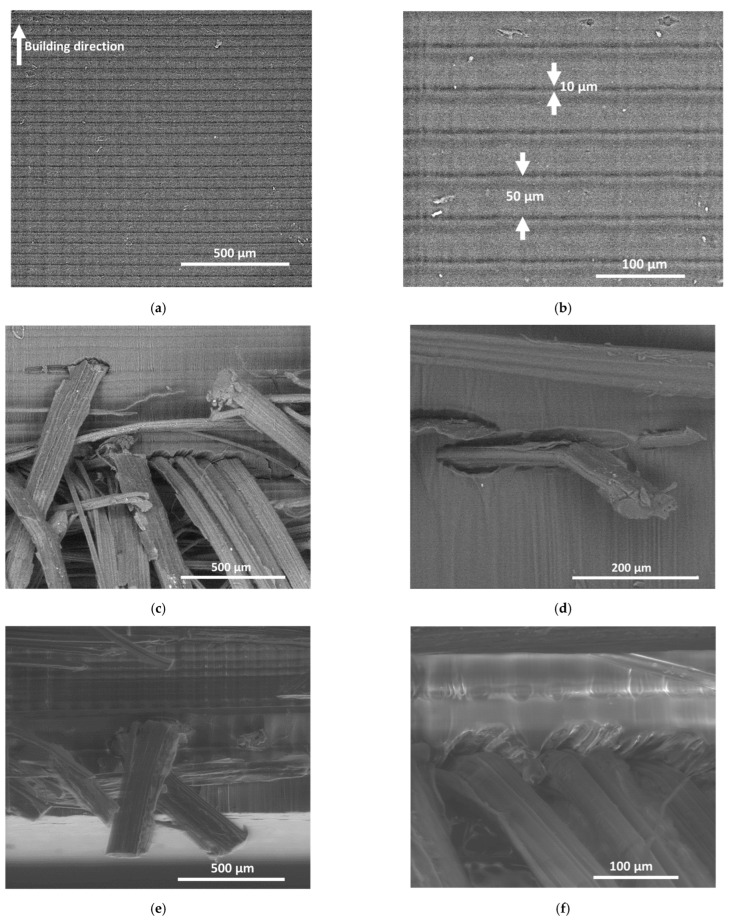
SEM micrographs of 3D-printed composites: (**a**) Cross-section view of 3D-printed resin with arrows showing the printing direction, (**b**) the same view with arrows embedded in the micrograph showing the necking and layer height, (**c**,**d**) Cross section views of the composite with hemp fiber reinforcement, (**e**,**f**) Views from the edge of the same composite under different magnifications.

To further explore the interpretation of the tensile results, the arrangement produced by the use of starch granules and natural fibers was studied. Figure 10 shows the SEM micrographs for LC00, LSC0, and LFC0 composites. Figure 10a provides a cross-section view showing the bulk arrangement along the building direction of the 3D-printed resin (LC00). At the macroscopic scale, the building direction cannot be discriminated, but the micrograph clearly shows a layering effect created by the DLP process similar to that observed in fused filament deposition. A closer view of the layers shown in Figure 10b demonstrates that between two successive layers, a minor spacing of approximately 10 µm alters the full cohesiveness of the resin. The average distance between two successive layers was equal to the prescribed layer height of 50 µm. Figure 10c represents a view perpendicular to the plane of construction near the fracture pattern. This view represents the material arrangement within the bulk of the composite. The layering of the resin was evident and consistent with the input layer height of 50 µm. Additionally, the clustering of the natural fibers with varied lengths and cross-sections is depicted in Figure 10c. A closer view of the fiber-matrix interface (Figure 10d) demonstrates the result of fiber pull-out, which is correlated to interfacial debonding. This debonding can explain the low tensile strength scores for LFC0, in comparison with those for other formulations. Figure 10e shows another zoomed-out view at the edge of the LFC0 composite. This view combines the cross-section view and the bottom surface view. This micrograph confirmed the absence of out-of-plane fibers on the surface of the composite, as suggested by visual examination of the composite. The entire hemp fiber population was within the bulk of the material, with only a few fibers closely oriented toward the top and bottom surfaces. The zoomed-in micrograph in Figure 10f also confirms the mechanism for fiber pull-out, which is still active during tensile loading close to the sample edges.

**Figure 11:**
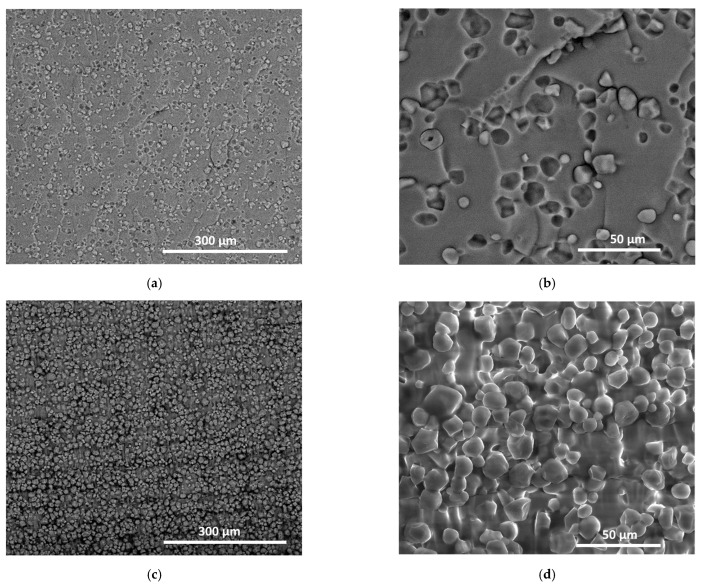
SEM micrographs of 3D-printed composites reinforced by starch granules. (**a**,**b**) surface view, and (**c**,**d**) bulk view.

Figure 11 shows the same micrographs for the case of starch filler according to two main plane views, the surface and cross-section views. Figure 11a suggests a homogeneous distribution of starch granules within the matrix. However, numerous pits are observed, which can be directly related to starch debonding at the fractured surface. The remaining starch granules were not found to be altered at the rupture point, which suggested that the prevailing failure mechanism also involved interfacial debonding. A closer view of the resin-starch interface (Figure 11b) shows no sign of transverse cracking at the surface, which indicates that a weak interface was formed between the starch granules and photosensitive resin. Additionally, there was an absence of a jagged surface outside the starch boundaries, which at a larger scale resulted in smooth fracture surfaces. This explains the low deviation with regard to the opening mode depicted in Figure 7c. The main difference between LFC0 and LSC0 that can explain the higher ranking of starch-based composite is the homogeneous arrangement of starch granules that is promoted by the starch-resin mixing in the liquid state. Figure 11c shows a bulky view of the material arrangement, where the layering effect can barely be observed, due to the large number of starch granules. This view also highlights the clustering of starch, but the density of the pits left by the starch granules detaching upon tensile loading was observed to be limited. A closer view confirmed this observation (Figure 11d), where the fracture pattern may be interpreted as a line of weakness running across the starch-starch interfaces.

## 4. Conclusions

This study showed the potential of using a mixture of photosensitive resin with natural fillers, such as hemp fibers and starch granules, to decrease the cost of feedstock materials and improve the environmental footprint of technical parts processed by additive manufacturing. DLP technology does not require the melting of starch, which is a significant problem in other technologies, such as fused filament deposition. DLP has led to successful attempts to print samples with a starch filler composition of up to 30%. The 3D printing process operated at room temperature with the help of photopolymerization did not alter the thermal stability of the fillers. The resulting tensile performance was found to depend primarily on the post-curing process, where the transition from an elastic—plastic material into a quasi-brittle material was observed for all composites. This demonstrated that the filler amounts considered in this study did not alter the photopolymerization process, irrespective of their shape and size. Only minor differences were observed between hemp-based and starch-based composites, which are explained by the relative homogeneity of starch-based composites. The most promising formulation, LSC0, resulted in a slightly improved stiffness compared with the reference photosensitive resin and the modulation of heat capacity with respect to the operating temperature.

## Figures and Tables

**Table 1:** Main characteristics of the photosensitive resin.

Property	Value (−)
Polymerization wavelength	405 nm
Viscosity (°25)	150–250 MPa.s
Color	transparent
Shore hardness	84D
Shrinkage (3D)	3.72–4.24%
Shrinkage (1D)	1.05–1.35%
Density	1.05–1.25 g/cm³
Flexural strength	59–70 MPa
Tensile strength	36–52 MPa
Elongation at break	11–20%

**Table 2:** Summary of printing conditions of photosensitive composites showing the weight proportion of each phase. LNG: longer photosensitive resin, STR: starch, FIB: natural fiber.

Condition	LNG (−)	STR (−)	FIB (−)	Curing (min.)
LG00	1	0	0	0
LC00	1	0	0	15
LSC0	2/3	1/3	0	15
LFC0	2/3	0	1/3	15
LSFC	1/3	1/3	1/3	15

**Table 3:** Thermal transition parameters of photosensitive 3D-printed composites; m_ech_ refers to the sample mass.

Materials	Thermal Transitions			
	Parameters	Peak 1	Peak 2	Peak 3
LC00 (m_ech_ = 29.1237 mg)	Enthalpy (J/g)	−203	26	23
	Onset (°C)	27	253	397
	Peak (°C)	67	290	413
	Endset (°C)	225	317	429
LSC0 (m_ech_ = 33.4995 mg)	Enthalpy (J/g)	−334	71	31
	Onset (°C)	27.5	291	393
	Peak (°C)	112	308	412
	Endset (°C)	254	337	435
LFC0 (m_ech_ = 26.8484 mg)	Enthalpy (J/g)	−211	36	31
	Onset (°C)	28	226	376
	Peak (°C)	59	280	399
	Endset (°C)	195	505	406

**Table 4:** Tensile properties for all studied composites.

Condition	e_F_ (−)	σ_T_ (MPa)	E_Y_ (MPa)
LG00	0.23 ± 0.00	24 ± 1	171 ± 7
LC00	0.07 ± 0.00	27 ± 1	417 ± 15
LSC0	0.06 ± 0.00	27 ± 2	451 ± 5
LFC0	0.07 ± 0.00	26 ± 1	399 ± 15
LSFC	0.07 ± 0.01	28 ± 4	400 ± 37

## Data Availability

Raw data are available from the authors upon request.

## Sample Availability

Not available.
